# Effects of Ball Milling Processing Conditions and Alloy Components on the Synthesis of Cu-Nb and Cu-Mo Alloys

**DOI:** 10.3390/ma12081224

**Published:** 2019-04-15

**Authors:** Xuekun Shang, Xitao Wang, Silian Chen

**Affiliations:** 1 Collaborative Innovation Center of Steel Technology, University of Science and Technology Beijing, Beijing 100083, China; xkshang0518@outlook.com (X.S.); xtwang@ustb.edu.cn (X.W.); 2 Central Iron and Steel Research Institute, Beijing 100081, China

**Keywords:** mechanical alloying, two-step ball milling, Cu alloy, immiscible alloy, SPEX milling

## Abstract

The effects of processing parameters in ball milling and the different behaviors of Cu-Nb and Cu-Mo alloys during milling were investigated. High powder yields can be obtained by changing the BPR value and ball size distribution and no clear dependence of BPR value on powder yield can be found from the experiment results. The addition of oxygen can largely reduce the effect of excessive cold welding during ball milling. A “two-step” ball milling method was introduced to evaluate the different evolution processes and morphologies in different alloys. With 8 h pre-milling, this method considerably benefits the oxidation process of Mo and shows its promising potential in the synthesis of immiscible alloys. Based on the experiment results and analysis, we suggest that the different behaviors of Cu-Nb and Cu-Mo alloys are related to the shear modules and different tendencies to be oxidized.

## 1. Introduction

Mechanical alloying (MA) is a well-known way in processing advanced materials involving repeated welding and fracturing the powder particles in a high energy ball mill [1,2]. First developed by Benjamin and his co-workers in 1970s [3,4], this processing method has been shown the potential in synthesizing supersaturated solid solutions, amorphous alloys, nano-crystalline, intermetallics, and oxide dispersion-strengthened alloys [5]. As a simple, elegant, efficient non-equilibrium processing method, MA is widely used to synthesis bulk supersaturated solid solutions used for many engineering applications. Large doses of energy are applied to powder particles during milling processing to produce nano-sized grains and supersaturated state of solute atoms in many alloy systems. However, the large amount of energy consumed when milling could be an obstacle in industrial application of this method [6]. Another problem is the low powder yield after MA, powder particles are sometimes easily get cold-welded to each other because of the heavy plastic deformation during milling, especially if they are ductile. Although a process control agent (PCA) can be used to reduce the final particle size and increase the powder yield, one need to consider the contamination level and possible interactions between the powders and the components in the PCA during the milling process [2,7,8,9,10,11,12,13]. Other than that, a number of process variables like milling time, milling speed, milling atmosphere, temperature of milling and the grinding medium also have effects on the final state of powder [14,15,16,17]. With all the milling parameters, MA is indeed a very complex process. Furthermore, all these process parameters are not completely independent, which makes it more difficult to increase the ball milling efficiency and to achieve the desired product phase or microstructure. Thus, many efforts have been made by investigators to get good understanding of the mechanisms related to the influences of processing parameters.

Ward et al. investigated milling process using different ball sizes and ball to powder mass ratios (BPR), introduced an expression for the milling dose to gauge the milling progress and found that the efficiency could remain constant over a range of BPR [16]. Razavi-Tousi et al. found that at a fixed ball-to-powder ratio, a change in ball size can significantly change the steady state milling time [6]. Gotor et al. studied the dependence of the ignition time (t_ig_) on the main parameters of the milling process such as spinning rate, powder charge, type and number of balls, established a direct relationship between the inverse of the ignition time and the power of the planetary mill [18]. Mojarrad et al. investigated the effect of filling ratio of vial (FRV) on the thermal behavior of Al-Fe_2_O_3_ thermite, showed the importance of FRV especially for mechanochemical synthesis and mechanically-activated combustion synthesis processes [19]. Broseghini et al. discussed the effect of jar shape on high-energy planetary ball milling efficiency and achieved enhanced comminution by suitably re-designing the jar shape [20]. Additionally, the effect of atmosphere and milling time were studied, Madavali et al. found that oxygen contamination played an important role in determining the final particle size and microstructure of the powder [21]. Similarly, Hegedűs et al. found the presence of oxygen obstructed the sticking of milled material and the milling medium, and the intrinsic strength of a metal determined the whether or not occurrence of spherical particles [22]. Caballero et al. also investigated the effect of ammonia and found that a short-time ammonia gas flow notably improved the hardness and strength of an Al-based alloy [23]. With more and more reports in literature, almost all the milling parameters have been studied to optimize the process, except that, we believe it is also worthwhile to consider the behaviors of different alloy components in milling. The mechanical or chemical properties of the milled materials could play a decisive role in ball milling.

In this work, we attempted to optimize the milling process by changing the ball size distribution and BPR. Copper oxide (CuO) powder was added into copper alloy system (Cu-Nb, Cu-Mo) not only to evaluate the influence of oxygen on the final state of powder particles, but also to obtain oxide dispersion-strengthened (ODS) copper alloys. A “two-step” ball milling method was introduced with the addition of CuO to illustrate the oxidation process of precipitates. Furthermore, we discussed the influence of different element properties on the behaviors of two alloy components (Nb and Mo) in mechanical alloying.

## 2. Materials and Methods

Powders of Cu (99.9% purity, APS 10 micron), Ag (99.9% purity, APS 4–7 micron), Nb (99.99% purity, APS 10 micron) and Mo (99.95% purity, APS 3–7 micron) purchased from Alfa Aesar (Ward Hill, MA, USA), were ball milled in a SPEX 8000D Dual Mixer/Mill^®^ (SPEX SamplePrep, Metuchen, NJ, USA). The SPEX shaker mill, which is most commonly used for laboratory investigations, operates by agitating a small grinding container with a capacity up to 5.5 × 10^−5^ m^3^ in three mutually perpendicular directions at approximately 20 Hz (1190 rpm) [1]. Stainless steel balls with three different sizes (6.35 mm, 9.525 mm, and 12.7 mm in diameter) were used in a hardened 440C stainless steel made grinding vial. Copper alloys with nominal atomic fraction Cu_85_Nb_5_Ag_10_ was investigated and the milling process was performed under argon atmosphere with different ball size distributions and BPR changing from 0.83 to 5.93 at room temperature. The addition of 10 at.% Ag prevented severe cold welding during milling and at the same time Ag powder acted as a marker for monitoring the deformation and mixing in the Cu lattice [24].

Furthermore, copper oxide (CuO) powder was added in the beginning of milling to introduce oxygen into the Cu-Nb/Cu-Mo alloy systems. All the to-be-milled powders were kept and loaded into the grinding vial inside a purified argon glovebox. After 10 to 30 h milling time, the milled powders were then collected and weighed in order to determine the powder yield. In addition, a “two-step” ball milling procedure was performed for comparison. The processing of the powders involved two steps: pure Cu, Nb/Mo and Ag powders were mixed and ball milled for 8 h to reach steady state after which Nb/Mo precipitates finely dispersed in copper matrix. CuO powder was then added at the beginning of the second step milling, powders were sampled after different interval of milling time (1, 2, 4, 6, 10, and 20 h). The nominal atomic fractions of the alloys are Cu_65_Nb_5_Ag_10_(CuO)_10_ and Cu_65_Mo_5_Ag_10_(CuO)_10_, respectively.

Subsequent to ball milling, an X-ray diffraction (XRD) instrument Rigaku MiniFlex-600 (Rigaku, Tokyo, Japan) with CuKα radiation (1.5406 Å) was used to obtain XRD patterns for phase determination, solubility and grain size measurement of the powders, with the range of 2θ from 25° to 60°, in a scan speed of 1°/min and a step width of 0.02°. The microstructures of some powder samples were characterized by scanning transmission electron microscopy (STEM) performed on a JEOL 2010F EF-FEG microscopes (JEOL, Ltd., Tokyo, Japan). STEM samples were prepared by FEI Helios 600 focused ion beam (FIB) (FEI Company, Hillsboro, OR, USA). 

## 3. Results and Discussion

### 3.1. Optimization of BPR and Ball Size Distribution

The BPR has a significant effect on the time required to achieve a steady state or particular phase in ball milling. Generally, a high BPR means a bigger mass or quantity of milling balls, thus increase the possibility of collisions to each other for the grinding medium. Consequently, more energy is used to accelerate the alloying process after transformed to the particles. Many related studies provided the BPR as a principal parameter to describe ball milling experiments. However, same BPR values can be obtained either by changing the number, size, or density of balls or changing the powder weight and balls at the same time. Milling was found to have different efficiency despite the same BPR [25], different ball size distributions at a fixed BPR significantly changed the steady state milling time [6]. The present work, however, is more concerned with the powder yields of ball milling at different BPR values and ball size distributions.

Table 1 gives the summary of the milling experiments performed on Cu_85_Nb_5_Ag_10_ alloy. Three different sizes of balls, referred to as S (small, 1/4 inch in diameter, 1.03 g), M (medium, 3/8 inch in diameter, 3.5365 g) and L (large, 1/2 inch in diameter, 8.355 g), were used in the milling to optimize the ball size distribution and BPR, the numbers before S, M, and L in column D are the quantity of used balls. All the ball milling experiments were performed for 10 h, interrupted every 2 h to check the status of the powder and cool down the vial. It should be noted that the powder yield data (with values greater than zero) in the table correspond to single experiments. Although we repeated the configuration in the sample 7 multiple times without recording the powder yields and those with zero powder yields for confirmation, it is an accepted fact that the alloying process among different powder particles happens when the balance between welding and fracturing achieved and reducing the effect of excessive cold welding is the key point to obtain good powder yield. Many investigators believe that a higher BPR value could result in more cold welding rather than fracturing and, thus, reduces the amount of powder produced. However, very different powder yields are achieved with similar (sample 2 and sample 5) or even the same (sample 3 and sample 4) BPR values as shown in Table 1. Both high and low BRP values can lead to good powder yields. Obviously, no clear dependence of BPR value on powder yield can be found from the experiment results. Perhaps the size and amount of balls have more influence on the powder yield in a small capacity, high-energy mill, such as the SPEX mill in which the BPR values are relatively low (<10:1). It was reported that the size of the grinding medium could affect the milling efficiency and the final constitution of powder [26,27,28,29]. Investigators ascribed it to the differences in the temperatures and the input energies of different grinding medium. Moreover, the use of different sizes of balls in the same experiment could minimize cold welding and thus the less powder coated onto the balls [30]. It was suggested that different sized balls will produce shearing forces and help to detach the powder on the balls. Thus, the powder yield could be higher with a combination of different sizes of balls. On the other hand, a combination of different sized balls helps to prevent a well-defined trajectory of moving balls. We manage to get high powder yields with different sized balls in sample 2 and sample 10, but we have an exception in sample 9. Therefore, it is complex to comprehend how the ball size difference affect the balance between welding and fracturing of powder particles, yet we suggest that the velocity change of balls and the hardness change of powder particles under different temperatures play an important role here.

Although no specific explanations have been given for the different powder yields under different ball size distribution and BPR values, it is possible for us to optimize the milling process by changing the size and amount of the grinding medium. We chose the configuration in sample 7 to perform the rest ball milling experiments for a relatively good powder yield and a suitable BPR value.

### 3.2. Synthesis of ODS Copper Alloy

#### 3.2.1. Addition of CuO

**Figure 1:**
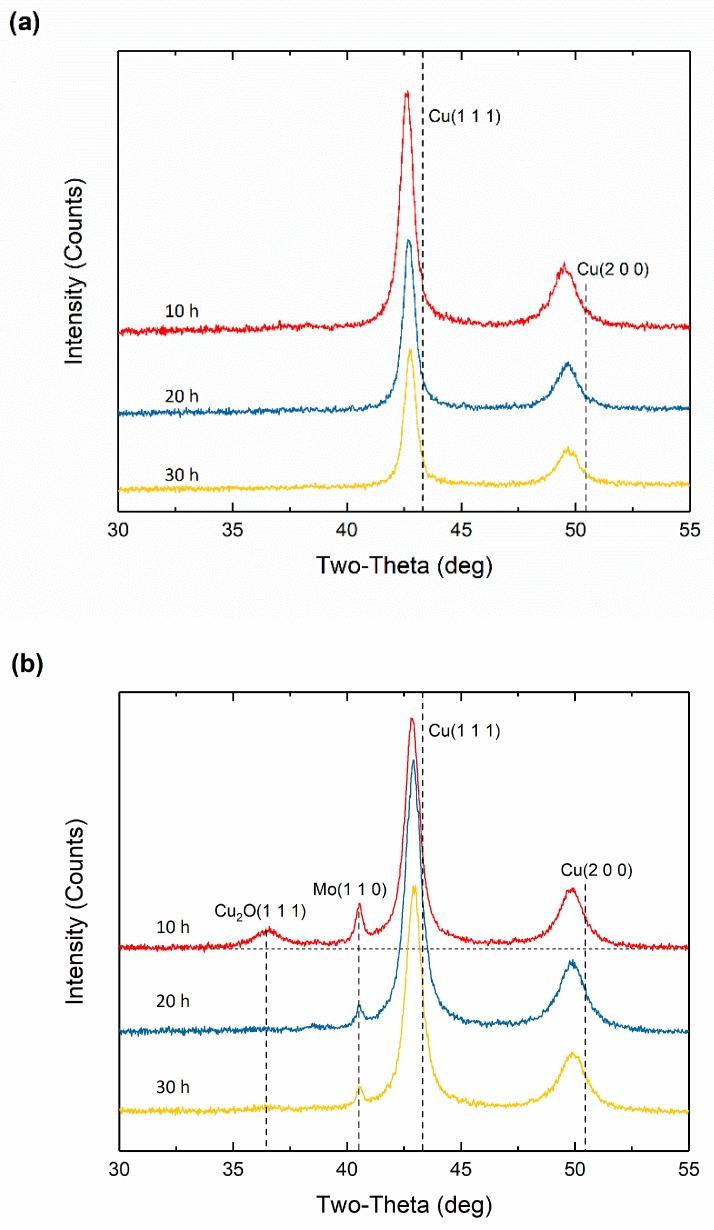
X-ray diffraction patterns of (**a**) Cu_65_Nb_5_Ag_10_(CuO)_10_ and (**b**) Cu_65_Mo_5_Ag_10_(CuO)_10_ alloys after 10, 20, and 30 h of milling.

Figure 1 shows the XRD patterns of Cu-Nb and Cu-Mo alloys after the addition of CuO at the beginning of milling. The powder diffraction file (PDF) database is used to identify the phases in the XRD patterns, PDF card numbers of each phase are listed in Appendix A. The broadening of reflections is caused by the reduction of grain size and the microstrains. Peak shifts to lower angles of Cu at (1 1 1) and (2 0 0) plane are noticed both in Figure 1a,b, indicating the dissolutions of Ag and possible Nb/Mo in copper matrix. For immiscible alloy systems with high heats of mixing like Cu-Nb and Cu-Mo, the solubilities are extremely low and the corresponding XRD peaks will exist even after a long time of high-energy ball milling [24,31]. However, with the help of CuO, a vanishing of Nb reflections can be observed after 10 h of ball milling, see Figure 1a. The calculation of the solubility of Nb was performed based on the lattice change by applying Vegard’s law and using the data of [32] for the lattice Cu-11.76 at.% Ag (assuming 75 Cu atoms and 10 Ag atoms in this alloy) as 3.6704 Å. The lattice parameters of Cu of 10 h, 20 h, and 30 h are 3.6770 Å, 3.6790 Å and 3.6686 Å in Figure 1a. The lattice parameter of Nb with fcc structure should be 4.1575 Å, which was calculated using molecular dynamics method. According to Vegard’s law, the solubilities can be obtained as 1.35%, 1.77% and −0.37%. Thus, Ag powder in matrix may not all in solution or part of the powder get lost when processing. Additionally, Vegard’s law is an empirical rule. Therefore, we find that it is difficult to figure out whether or not can the integration of Nb/Mo affect the Cu diffraction shift. It is possible that the oxygen extends the solubility of Nb here, however, it cannot be the only reason of the total vanishing of Nb in XRD patterns; otherwise, a larger shift of Cu diffraction would happen if most of the Nb get into solution. The reaction between oxygen and Nb may account for this though no evidence of Nb oxide existence is found in the X-ray diffraction patterns. In fact, Kim et al. revealed that nano-sized Ti particles in Al matrix were not detected by XRD method and peak disappearance of the minor phase did not reflect the formation of true solid solution [33]. Therefore, nano-sized Nb oxide could possibly formed during the Cu_65_Nb_5_Ag_10_(CuO)_10_ ball milling considering the detection limit of XRD method. Figure 1b, however, shows a quite different behavior of Mo during ball milling. Peaks of Mo in XRD pattern are found even after milling for 30 h despite the addition of CuO, indicating a certain portion of Mo powder is not oxidized by the oxygen from CuO powder. Although with the intensity reduction of diffraction peaks, it is assumed that Mo have been progressively consumed by oxygen. Notably, a new phase Cu_2_O can be found in the system after 10 h of milling and disappears before the milling time reaches 20 h. CuO is believed to have reacted with Cu to form Cu_2_O in the first 10 h. Generally, a high temperature is needed to initiate this reaction which, on the other hand, shows an advantage of ball milling for making some reactions happen more easily. In this case, Nb is obviously more easily oxidized than Mo and it seems that the CuO powder would rather react with Cu than Mo. After 30 h of ball milling with the addition of CuO, the two elements (Mo and Nb) show different phase evolution processes and final states probably due to their different chemical properties. In order to make a better observation of alloying process during ball milling after the addition of CuO, we performed a two-step ball milling procedure.

#### 3.2.2. Two-Step Ball Milling

CuO powder was added into the milling vial after the Cu, Nb/Mo and Ag powders were pre-milled for 8 h. Powder specimens (less than 0.2 g each) were taken from the same milling vial after different interval of time in the second milling step with a total time of 20 h. We could obtain over 9 g of fine powder from a total 10 g of powder added into the vial. The slightly increase of BPR value due to the mass of specimens shouldn’t affect the high-energy milling process much as we discussed above. What is notable here is that we can always get a high powder yield (over 90%) after the addition of CuO powder. It seems that the present of oxygen can reduce the effect of excessive cold welding which is consistent with what Madavali et al. [21] and Hegedűs et al. [22] found in their studies.

**Figure 2:**
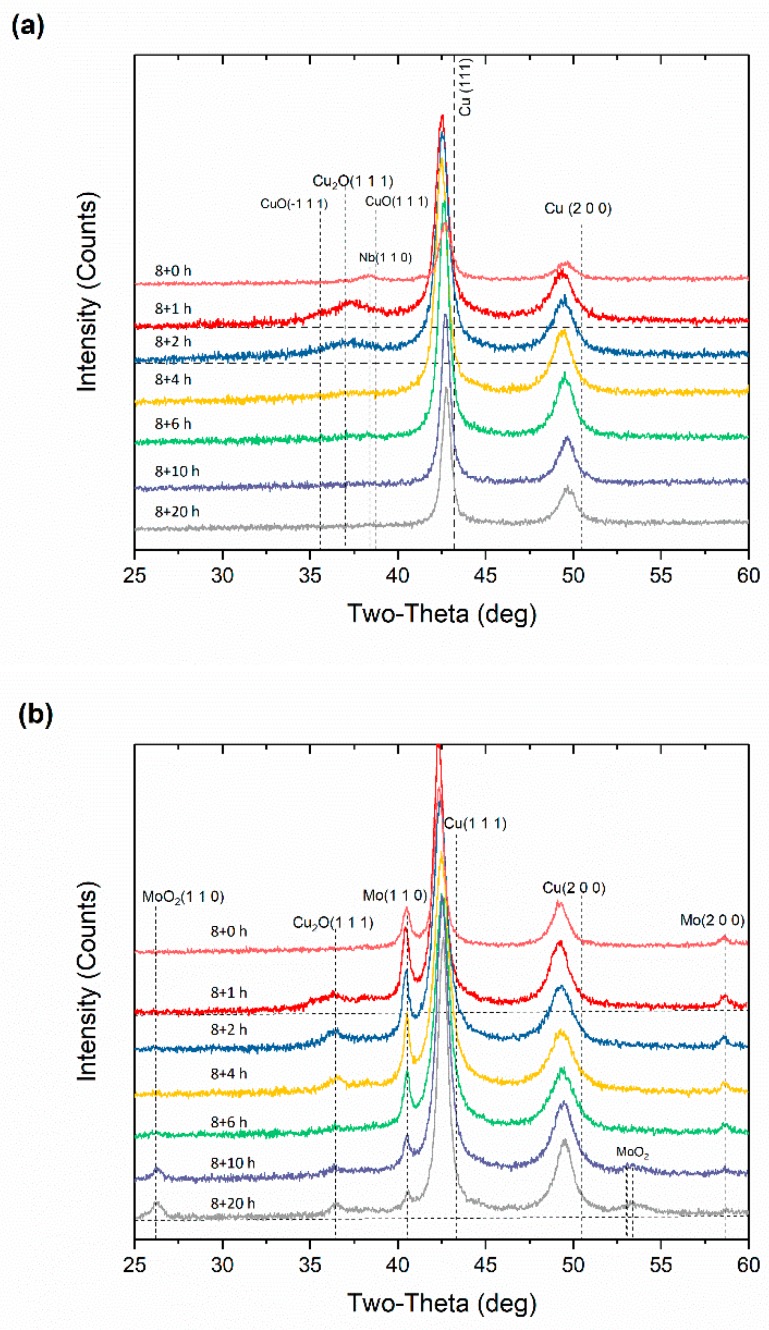
X-ray diffraction patterns of (**a**) Cu_65_Nb_5_Ag_10_(CuO)_10_ and (**b**) Cu_65_Mo_5_Ag_10_(CuO)_10_ alloys as a function of second-step milling time.

Figure 2 depicts the X-ray diffraction patterns obtained from the powder specimens with varying second-step milling times. The different values after “8+” are the milling times of the second step. Again, the peak shifts to lower angles of Cu and the broadening of peaks are observed; the disappearance of Nb peaks and the existence of Mo peaks with the addition of CuO are shown in Figure 2a,b. One of the differences in Figure 2 is that the peaks of Mo oxide (MoO_2_) are obvious in XRD patterns after milling for 10 h in second step, unlike what is shown in Figure 1b. Thus, we can confirm the existence of Mo oxide precipitates and we believe that a “two-step” ball milling procedure makes the oxidation process of Mo easier with finely dispersed Mo precipitates in copper matrix after 8 h milling in first step. Note that Cu_2_O phase forms in both Nb and Mo alloy at the beginning of the second-step milling. However, peaks of Cu_2_O disappear before 4 h in Figure 2a and exist after 20 h in Figure 2b. This suggests that the oxidation process of Nb is easier and faster than that of Mo.

**Figure 3:**
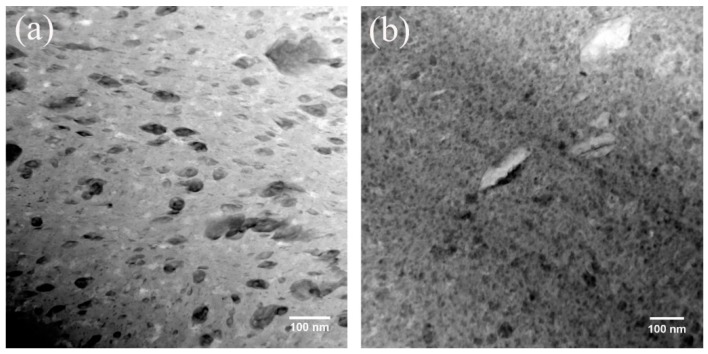
HAADF images of (**a**) Cu_65_Nb_5_Ag_10_(CuO)_10_ alloy ball milled for (8 + 4) h; and (**b**) Cu6_5_Mo_5_Ag_10_(CuO)_10_ alloy ball milled for (8 + 20) h.

The high-angle annular dark field (HAADF) images of Cu-Nb and Cu-Mo alloys after milling are shown in Figure 3. Although with no signs of Nb oxide in Figure 2a, the images in Figure 3 reveal the existence and the morphology of both Nb and Mo oxide precipitates. As Z-contrast imaging is used in HAADF images, the intensity in imaging is proportional to the square of the average atomic number, suggesting the dark precipitates should be Nb/Mo oxide precipitates. Energy dispersive spectroscopy (EDS) results also show that the dark regions are rich in Nb/Mo and oxygen. Note that the Mo oxide precipitates are uniformly dispersed in Cu matrix with a narrow size distribution as shown in Figure 3b. However, the Nb oxide precipitates in Figure 3a are mainly composed of two types of precipitates with different sizes, smaller type less than 10 nm and the other around 20–50 nm. Except the different tendencies to be oxidized, we suggest the large difference of the shear modules of Nb (38 Gpa) and Mo (120 Gpa) could also be related to the different behaviors of these two alloys. Since the shear modulus is proportional to the Peierls-Nabarro stress, which is the force needed to move a dislocation, the Nb precipitates are easier to be cut by dislocations during deformation. This process introduces more interfaces, which makes more diffusion between elements, and together with a higher tendency to be oxide in Nb alloy as we described before, explain the faster oxidation process of Nb compared with Mo. It takes a longer time before the reaction between Cu_2_O and Mo particles, because of the low diffusivity of Mo, probably the size of both Mo and Cu_2_O particles should be small enough before the formation of Mo oxide precipitates, leading to a homogeneous morphology. The larger precipitates in Figure 3a are possibly transformed from deformed Cu_2_O particles which react with Nb before the size being reduced to below 20 nm by ball milling. The Nb in solution is supposed to precipitate out from Cu matrix and then be oxidized, resulting in small precipitates (<10 nm) in Figure 3a. It can, therefore, be concluded that the different evolution processes and morphologies of Mo and Nb alloys during ball milling are related to their different tendencies to be oxidized and shear modules.

## 4. Summary and Conclusions

The influences of processing parameters on mechanical alloying were discussed and investigated in this study to optimize this synthesis method. Ball milling experiments on Cu-Nb and Cu-Mo alloys were performed at room temperature with the addition of CuO powders and a “two-step” ball milling method was introduced in order to evaluate the different evolution processes and morphologies in different alloy systems. We discussed how the chemical and mechanical properties of different elements affect the ball milling process and the final state of milled powder. Based on the results and previous analysis, the following conclusions can be drawn:

1. High powder yields can be obtained by changing the BPR value and ball size distribution.
2. No clear dependence of BPR value on powder yield can be found from the experiment results.
3. The addition of oxygen can largely reduce the effect of excessive cold welding during ball milling.
4. A “two-step” ball milling procedure considerably benefits the oxidation process of Mo and shows its promising potential in the synthesis of immiscible alloys.
5. The chemical and mechanical properties of elements play a critical role in the evolution process and morphologies of different alloys during milling.

## Figures and Tables

**Table 1:** Summary of the milling experiments performed on Cu_85_Nb_5_Ag_10_ alloy. N is the label of the sample in each run. *M_a_* is the mass of the powder added into the milling vial. *M_b_* is the mass of the powder recovered after mill (measured). D is the detail about the size and quantity of balls used in a run. BPR is the ball to powder mass ratio. P is the powder yield of a run (=$\frac{M_{b}}{M_{a}}\times 100\%$).

N	D	*M_a_*	BPR	*M_b_*	P
1	1L	10 g	0.8355	5.85 g	58.5%
2	1S1M	5 g	0.912	3.76 g	75.2%
3	2M	10 g	0.7073	2.83 g	28.3%
4	3M	15 g	0.7073	0	0
5	3M	10 g	1.059	0	0
6	4M	10 g	1.41	0	0
7	7M	10 g	2.47	4.65 g	46.5%
8	7M	5 g	4.95	1.55 g	31%
9	1L1M	10 g	1.19	0	0
10	1L2M	10 g	1.54	5.93 g	59.3%

## Appendix A

The PDF data used for the identification of phases in the XRD patterns are as follows: Cu–PDF#01-085-1326; Nb–PDF#04-012-8010; Mo–PDF#04-001-0059; Ag–PDF#04-001-2617; CuO–PDF#04-007-1375; Cu_2_O–PDF#04-007-9767; NbO_2_–PDF#04-001-1744; MoO_2_–PDF#01-086-0135.
